# Validity and reliability of the Chinese version of the Normalization MeAsure Development(NoMAD)

**DOI:** 10.1186/s12913-022-08737-1

**Published:** 2022-11-11

**Authors:** Mengyao Jiang, Qing Wang, Tracy Finch, Dongli She, Yujun Zhou, Yuet Foon Chung, Jie Chen, Lin Han

**Affiliations:** 1 Evidence-Based Nursing Center, School of Nursing, Lanzhou University, Chengguan District, No.28 Yanxi Road, Lanzhou, Gansu, 730000 China; 2 Department of Nursing, Tongji Hospital, Tongji Medical College, Huazhong University of Science and Technology, Wuhan, China; 3 Department of Nursing, Midwifery and Health, Northumbria University, Newcastle Upon Tyne, UK; 4 Department of Nursing, Gansu Provincial Hospital, Lanzhou, Gansu, China; 5 School of Nursing, University of Connecticut, Storrs, CT USA

**Keywords:** Normalization process theory, NPT, Implementation, Psychometric properties, Pressure injury, Nurse

## Abstract

**Background:**

The Normalization MeAsure Development (NoMAD) is a brief quantitative tool based on the Normalization Process Theory (NPT), which can measure the implementation process of new technologies and complex interventions. The aim of our study was to translate and culturally adapt the NoMAD into Chinese, and to evaluate the psychometric properties of the Chinese version of NoMAD.

**Methods:**

According to the NoMAD translation guideline, we undertook forward translation, backward translation, and compared these translations to get a satisfactory result, then we performed cognitive interviews to achieve cross-culture adaptation. And the psychometric properties of the final version were evaluated among clinical nurses who used the pressure injuries management system via WeChat mini-program at a tertiary hospital in northwestern China.

**Results:**

A total of 258 nurses were enrolled in our study, and the response rate was 92.1%. The Cronbach’s alpha of four dimensions were as follow: Coherence (0.768), Cognitive Participation (0.904), Collective Action (0.820), and Reflexive Monitoring (0.808). The overall internal consistency was 0.941. The confirmatory factor analysis results showed a good fit for its theoretical structure (CFI = 0.924, TLI = 0.910, RMSEA = 0.0079, SRMSR = 0.046, χ^2^/df = 2.61). The item-level content validity index ranged from 0.857 to 1, and the scale-level content validity index was 0.95. There were positive correlations between four constructs scores and three general normalization scores.

**Conclusions:**

The Chinese version of NoMAD is a reliable and valid tool to evaluate the implementation process of innovations.

## Background

Implementation research can address the challenges of the substantial gap between research evidence and clinical practices, which is increasingly paid attention to by researchers in recent years [1–4]. In the field of implementation science, Proctor and colleagues proposed eight core implementation outcomes including acceptability, adoption, appropriateness, feasibility, fidelity, implementation cost, penetration and sustainability [5]. Sustainability emphasizes the routinization and institutionalization of innovations. Based upon results from a recent systematic review of the implementation outcome instruments [6], there are only two instruments to assess sustainability, one is designed for long-term care [7], of which reliability and validity were poor, and the other one is the Normalization MeAsure Development (NoMAD).

NoMAD is a simple quantitative tool to evaluate factors that promote or inhibit implementation success from the perspective of participants [8], which has been adapted into Swedish [9], Dutch [10] and Brazilian Portuguese [11]. The original NoMAD instrument has been applied to a diverse range of interventions, such as a health literacy training program [12], a shared decision-making conversation tool [13], telehealth [14], surgical safety checklist [15], pharmacy workforce [16], a dietitian-led model of care [17], weight management program [18], a novel oral health risk assessment tool [19] and the primary care services for transgender individuals [20]. It was developed by Finch et.al in 2018, through item development, item testing and developing indicators [21, 22]. NoMAD was derived from the Normalization Process Theory (NPT) which aims at describing and explaining the implementation, embedding, integration of organizational innovations and complex interventions into clinical routine [23, 24].

### Introduction of pressure injuries management system

Pressure injury (PI) is defined as “localized damage to the skin and/ or underlying tissue, as a result of pressure or pressure in combination with shear”. PI usually occurs over a bony prominence but may also be related to a medical device or other objects. PI is an indicator of the quality of care and is a common and significant clinical concern among nurses [25–27]. The current PI management relies on paper-based medical records or computer-based information system, which is inefficient and time-consuming. Fortunately, with the evolution of information technology, mobile health (mHealth) has been increasingly used in clinical practice [28, 29]. WeChat application is the most popular social media platform in China, with more than one billion active daily users [30]. Mini program is a sub-application embedded in WeChat. There is no need for downloading, installing, registering, or logging in mini-program, which is easy to use and saves space for the smartphone. People can get into a mini-program via scanning a QR-code or social sharing.

The “Longhuhui” PI management system via WeChat mini-program was developed by the nursing department of a 2600-bed tertiary academic teaching hospital in Northwestern China. The PI management system was mainly used for hospitalized patient PI risk assessment (based on the Braden scale), which aimed to replace the traditional paper version of the PI risk assessment form. In addition, for PI high-risk patients, the preventive interventions would be recorded; for patients with PI, the wound assessment and interventions would be recorded. The system was officially put into use in the whole hospital on January 1st, 2021. The WeChat account of each clinical nurse was registered as a real-name account and only certified nurse accounts can log into the PI management system. The PI management of the hospital was a three-level quality management system, and each department was equipped with one or two PI coordinators. The charge nurses assess the PI risk of newly admitted patients; if the patient had PI, it would be reported by the PI coordinators. All risk assessments and wound assessments would be reviewed by the head nurse and then reported to the quality control team of the nursing department.

### Aim

The aim of our study was to translate and culturally adapt the NoMAD into Chinese, and to evaluate the psychometric properties of the Chinese version of NoMAD by testing PI management system via WeChat mini-program among Chinese nurses.

## Methods

### Study design

Our study used a mixed-methods approach to develop and validate the Chinese version of NoMAD (C-NoMAD), following a four-phase process [31]. This process includes translation, cross-cultural adaption, pilot test and psychometric testing.

### NoMAD instrument description

The English NoMAD instrument consists of three parts: part A is about participant’s information; part B has three general questions about participant’s experience and acceptability of intervention in three different perspectives, using a 10-point Likert scale, with higher scores indicating better normalization; and part C has 20 items based on NPT four core constructs: Coherence (4 items), Cognitive Participation (4 items), Collective Action (7 items), and Reflexive Monitoring (5 items), this part has two options, option A is a 5-point Likert scale (strongly agree to strongly disagree); option B is the irrelevant reason (not relevant to my role/at this stage/to the intervention). The English version of NoMAD is freely available under creative commons licence for research purposes [32].

### Phase 1 translation

According to the NoMAD translation guideline [31], forward translation was conducted by two bilingual speakers (Chinese as the native language) independently, backward translation was conducted by two native English speakers who had not seen the original NoMAD instrument. Then, the researcher team members compared the translations and discussed the appropriate translation draft. The comparability between the translations was often the different expression of synonyms, such as “feel → think, affect → influence, people → personnel, legitimate → legal, be open to → be willing to, adequately → fully, effect → impact”.

### Phase 2 cross-cultural adaption

To understand whether the tool developed in the English context can be generalized to the Chinese cultural context and to ensure the equivalence of the tool measurement, we conducted a face-to-face interview using the C-NoMAD translation draft. Through convenience sampling, we selected ten healthcare workers to investigate their understanding and perspective about each C-NoMAD item. The inclusion criteria of participants were frontline clinical staff who had used mHealth or eHealth in clinical work and voluntarily participate in this study.

In an interview, firstly, the researcher explained the purpose and process of the study; secondly, asked the interviewees to sign the interview informed consent form; thirdly, asked them to fill in the C- NoMAD translation draft; then, the researcher interviewed them according to the interview guide, Table 1 shows the list of open-ended questions or prompts. If the interviewees raised questions about the expression of some items, the researcher would explain the meaning of the original items, then the interviewees were asked to provide appropriate expressions according to their own understanding. Interviews took place in an undisturbed room at the hospital and lasted between 45 and 60 min. All interviews were audio-recorded, meanwhile, the researcher took the corresponding notes. Based on the interview results, the expressions of some items were revised and a tentative draft of the C-NoMAD was formed after the discussion of the researcher team. The adjustment was about the subtle expression of Chinese.

**Table 1:** Interview questions

**1. General comprehension**
• How do you understand the C-NoMAD survey purpose?
• Is there any item hard to understand? If there is, could you please tell me the reason and provide a better expression?
• How do you understand normalization?
**2. Instruction comprehension**
• How do you understand the meaning of option A and option B?
• Please explain the differences among “strongly disagree” “disagree” “neutral” “agree” “strongly agree”
• Please explain how do you understand “not relevant to my role” “not relevant in this stage” “not relevant to the intervention”?
**3. Item comprehension**
• How do you understand the difference of these three questions in Part B?
• Please see the item #, how do you understand the meaning of the item # “XXX”, which option do you choose? Why?

### Phase 3 pilot test

The inclusion criteria of experts were being specialized in psychometrics or implementation science or evidence-based medicine, having the title of associate professor or above, and having a Master's degree or above education background.

The expert consultation questionnaire consisted of three parts: (1) Letter to experts: introduction about the purpose and content of the study, the background of NPT and NoMAD; (2) Expert basic information form: demographic information of the experts, their familiarity with the research questions, and the basis of their judgment; (3) Expert evaluation form: asking the experts to choose the relevance or representativeness of each item of the NoMAD tool concerning the corresponding content dimension, with options for a four-level rating (1 = not relevant, 2 = relevant, 3 = relatively relevant, 4 = very relevant), as well as comments on modifications to each item and overall suggestions for this study.

Based on the inclusion criteria, the researcher identified seven appropriate experts. For experts in Lanzhou City, the researcher introduced the purpose of this study and relevant background information to the expert in person before issuing a paper version of the expert consultation questionnaire. For experts outside Lanzhou city, the researcher contacted them by e-mail which included the electronic version of the expert consultation questionnaire and the original version of the NoMAD tool.

After collecting expert consultation questionnaires, we summarized and synthesized all experts’ comments on item modifications. Then, according to the experts’ comments, the research team members discussed and modified some items to form the final translation of the Chinese version of the NoMAD instrument.

### Phase 4 psychometric testing

The researchers came to clinical departments in person and in each department first invited the head nurse to fill in the paper-based questionnaire, and asked if any items were difficult to understand. If so, the researcher would explain and record the problematic items. After that, the head nurse distributed them to the department nurses to fill in, and the researcher then went to the department at the appointed time to collect the questionnaires. The survey questionnaire consists of three parts: (1) demographic information including age, sex, working years, educational level, position, professional title and working department; (2) the 23-item Chinese version of the NoMAD instrument; (3) two open-ended questions “Is there any item hard to understand? if there is, please write down the item number and its reason.” and “Do you have any suggestion for improving the PI WeChat mini-program implementation?”. The inclusion criteria of participants were clinical nurses who had used the PI WeChat mini-program and volunteered to participate in our study. The exclusion criteria were nurses working less than one year in the clinic, considering they were still in a standardized training period who had not managed the patients independently and were not familiar with the mini-program. To conduct the structural equation model, a minimum of 200 participants were required [33].

### Statistical analysis

We used descriptive analysis to summarize participants’ demographic information. The COnsensus-based Standards for the selection of health Measurement INstruments (COSMIN) guided our outcome measures [34].

For reliability, we used Cronbach’s alpha coefficient to examine internal consistency. The cut-off value is equal to or greater than 0.70 was considered acceptable [35].

For validity, we tested the C-NoMAD by assessing two aspects: content validity and construct validity. Content validity was calculated by content validity index (CVI), including Item-level CVI (I-CVI) and scale-level CVI (S-CVI) [33]. The value of I-CVI and S-CVI equal to or greater than 0.8 was considered good. We used confirmatory factor analysis (CFA) by the robust Weighted Least Square Means and Variances (WLSMV) estimator to assess construct validity [36]. According to the recommended cutoff criteria, the adequate model fit was defined as the value of Tucker Lewis Index (TLI) and comparative fit index (CFI) are greater than 0.9, respectively [37]; and the value of the root mean square of approximation (RMSEA) and standardized root mean square residual (SRMR) are less than 0.08, respectively [38].

We also calculated bivariate correlations between three general questions scores and four construct and overall scores via Pearson correlation coefficients.

We used IBM SPSS for Windows (Version 25.0 Armonk, NY, USA) and AMOS to perform all statistical analysis.

### Ethical considerations

Our study protocol was approved by the ethics committee of nursing school of Lanzhou University (LZUHLXY20190062). Before commencing each face-to-face interview, verbal consent was obtained from all interviewees. Prior to the start of data collection, all participating nurses were informed of the details about the study and signed written informed consent.

## Results

### Characteristics of study participants

A total of 280 questionnaires were collected in the survey, of which 22 were missing data and answered regularly, so the number of valid questionnaires was 258, and the response rate was 92.1%. The majority of the survey respondents were female (96.9%), senior nurse (60.5%), bachelor’s degree (95.0%). The average ages of the participants were 32.48 ± 6.30 years, ranging from 21 to 57 years, and the average working years were 9.66 ± 7.34 years, ranging from 1 to 36 years. The detailed information sees Table 2.

**Table 2:** Characteristics of participants (*n* = 258)

	Number	Percent
**Sex**		
Female	250	96.9%
Male	8	3.15
**Educational level**		
Junior college or below	8	3.1%
Bachelor	245	95.0%
Master or above	5	1.9%
**Position**		
Ordinary nurse	203	78.7%
PI coordinator	28	10.9%
Wound specialist nurse	5	1.9%
Head nurse	22	8.5%
**Professional title**		
Junior nurse	33	12.8%
Senior nurse	156	60.5%
Supervisor nurse	60	23.2%
Vice director and above	9	3.5%
**Department**		
Medical	89	34.5%
Surgery	92	35.7%
ICU	25	9.7%
Gynecology and pediatrics	13	5.0%
Others ^a^	39	25.2%

^a^refer to the geriatrics department, traditional Chinese medicine department, rehabilitation medicine department and infectious diseases department

### PI management system implementation results

**Fig. 1:**
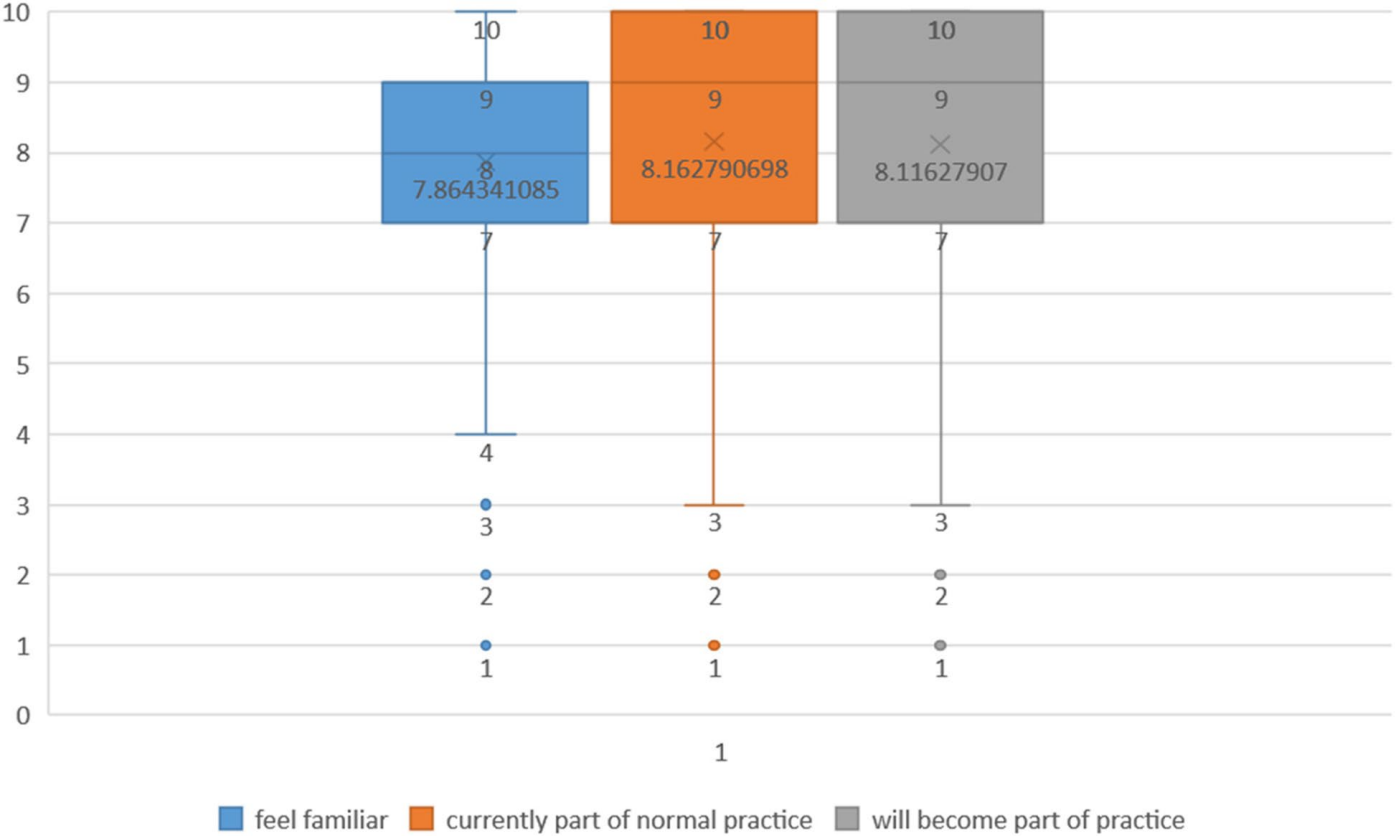
Results of C-NoMAD three general questions (*n* = 258)

Figure 1 shows the results of three general questions. Table 3 presents the participants’ responses to each item of Part C. The majority of responses were chosen “agree” or “neutral”. According to the participants’ feedback, the most hard-to-understand item was item #3 “I understand how the [intervention] affects the nature of my own work”.

**Table 3:** Participants’ response to the PI management system implementation (*n* = 258)

	Strongly agree	Agree	Neutral	Disagree	Strongly disagree	Not relevant to my role	Not relevant at this stage	Not relevant to the intervention
1.I can see how the [intervention] differs from usual ways of working	44	140	59	9	4	1	0	1
2.Staff in this organisation have a shared understanding of the purpose of this [intervention]	35	100	83	33	5	1	1	0
3.I understand how the [intervention] affects the nature of my own work	41	139	54	17	5	0	2	0
4.I can see the potential value of the [intervention] for my work	34	106	90	24	2	0	2	0
5.There are key people who drive the [intervention] forward and get others involved	61	132	50	12	0	2	1	0
6.I believe that participating in the [intervention] is a legitimate part of my role	53	145	43	14	2	0	0	1
7.I am open to working with colleagues in new ways to use the [intervention]	52	127	56	17	5	0	0	1
8.I will continue to support the [intervention]	52	141	47	15	2	1	0	0
9.I can easily integrate the [intervention] into my existing work	32	118	63	38	5	1	1	0
10.The [intervention] disrupts working relationships	20	68	91	55	22	1	1	0
11.I have confidence in other people’s ability to use the [intervention]	29	100	100	24	2	2	1	0
12.Work is assigned to those with skills appropriate to the [intervention]	30	109	64	47	6	1	1	0
13.Sufficient training is provided to enable staff to use the [intervention]	52	148	42	14	1	0	0	1
14.Sufficient resources are available to support the [intervention]	51	135	46	23	1	1	0	1
15.Management adequately support the [intervention]	73	146	29	7	1	1	0	1
16.I am aware of reports about the effects of the [intervention]	32	92	88	33	5	3	4	1
17.The staff agree that the [intervention] is worthwhile	37	104	72	36	8	1	0	0
18.I value the effects the [intervention] has had on my work	30	125	82	15	4	1	1	0
19.Feedback about the [intervention] can be used to improve it in the future	47	138	59	9	2	1	1	1
20.I can modify how I work with the [intervention]	30	129	71	20	7	0	1	0

According to the feedback, no one filled in the first open-ended questions about the problematic items. A total of 49 people had written about the PI management system suggestion. The main comments can be grouped into 3 topics: (1) the proposal on the design of PI management system (26, 53.1%); (2) the standpoints on increasing clinical workload after the PI management system implementation (21,42.9%); (3) the recommendations on conducting more relevant training (2, 4%).

### Internal consistency reliability

The overall Cronbach’s alpha coefficients of the C-NoMAD was 0.941, and that of the four dimensions were as follows: Coherence (0.768), Cognitive Participation (0.904), Collective Action (0.820), and Reflexive Monitoring (0.808). The results indicate that C-NoMAD has good internal consistency.

### Content validity

The results of content validity analysis show that the S-CVI was 0.95, which is above the recommended level of 0.8. And the I-CVI ranged from 0.857 to 1, except for the I-CVI of item #3 was 0.714.

### Construct validity

**Fig. 2:**
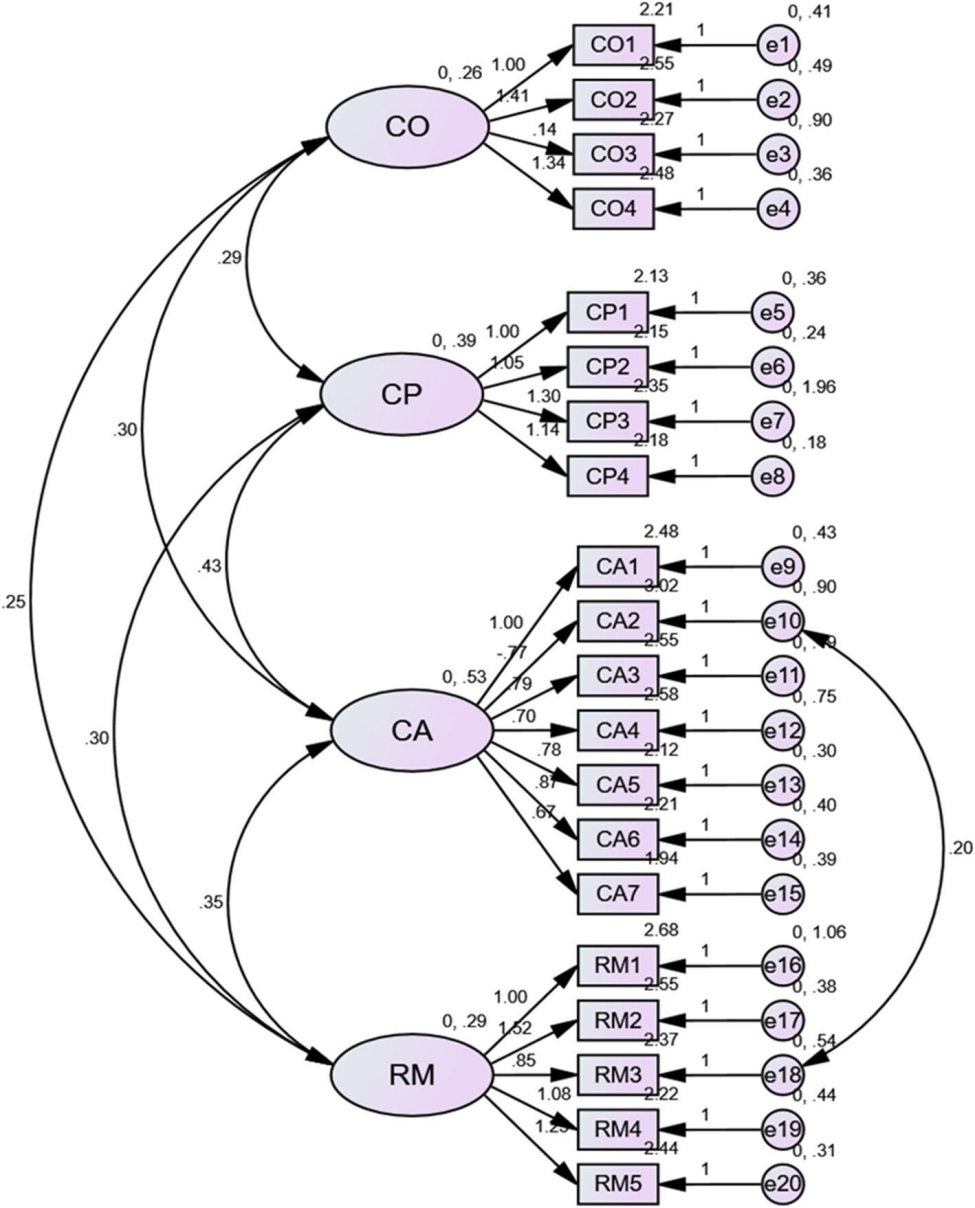
Results of C-NoMAD four core constructs factor loadings (*n* = 258)

Using AMOS 25.0 software, we verified the fit of the C-NoMAD to the NPT model. The CFA results showed that the factor loading of item #10 was less than 0.5. When unmodified, the RMSEA did not reach the recommended value. According to the hint of the modification indices, we set item #10 and #18 as error correlations. After being modified, all the fit indices reached the recommended value which means the C-NoMAD has acceptable construct validity (Table 4). However, item #3 “I understand how the [intervention] affects the nature of my own work” still had the lowest factor loading. Figure 2 presents the standardized factors loadings of C-NoMAD.

**Table 4:** Results of C-NoMAD construct validity (*n* = 258)

	χ^2^	df	χ^2^/df	RMSEA	SRMR	CFI	TLI
Unmodified	397.818	146	2.725	0.082	0.0484	0.918	0.904
Modified	378.800	145	2.612	0.079	0.0465	0.924	0.910
Recommended	-	-	< 5.0	< 0.08	< 0.08	> 0.9	> 0.9

### Correlations

Table 5 shows the positive correlations between four constructs’ scores and overall scores and three general questions scores. The Pearson correlation coefficients ranged from 0.251 to 0.476 (*P* < 0.01), which means their correlations were low to moderate.

**Table 5:** Correlations between three general questions scores and four construct and overall scores (*n* = 258)

	Familiar	Currently	Future
Coherence	0.374	0.301	0.408
Cognitive Participation	0.341	0.370	0.462
Collective Action	0.340	0.368	0.430
Reflexive Monitoring	0.251	0.283	0.339
overall	0.372	0.385	0.476

*P* < 0.01

## Discussion

Our study translated the English NoMAD instrument into Chinese and performed the psychometric evaluation of the C-NoMAD in the context of the implementation of the pressure injuries management system via WeChat mini-program. The C-NoMAD is a brief quantitative tool to measure implementation sustainability for Chinese researchers.

There are some questionable items needed to be discussed. Item #3 “I understand how the [intervention] affects the nature of my own work”, the word “nature” in Chinese is a very abstract concept, our investigation shows that quite a lot of clinical professionals propose this item was hard to catch its meaning, although we tried many attempts to make it more understandable. Item 10 “The [intervention] disrupts working relationships.”, many participants asked the “working relationships” means which one relationship, nurse-to-nurse relationship or nurse-to-patient relationship, and so on. Item #12 “Work is assigned to those with skills appropriate to the [intervention].” The initial goal to develop the PI management system via WeChat mini-program is easy to operate and requires every clinical nurse in our study hospital can use it, so there is totally no working assignment problem.

For reliability, the internal consistency of C-NoMAD shows greater results than the other language version of NoMAD. The reason may be related that our study was only conducted in one hospital and assessed one intervention, and the other version assessed several interventions in different settings (e.g. the English version tested six interventions in six sites, and the Dutch version assessed one intervention in three groups).

For validity, the CFA shows that the C-NoMAD has an acceptable fit for NPT theoretical four-construct model after being modified. Item #3 had the lowest factors loading which is consistent with our interview result. And the C-NoMAD has good content validity, except for item #3. Accordingly, in the final version of C-NoMAD, we decided to delete item #3. The problem about item #3 had never been mentioned by the other language version of NoMAD. English, Swedish, and Dutch are Indo-European family of Germanic languages, however, Chinese belongs to Sino-Tibetan family of Chinese languages, the language family is different. Moreover, it may be affected by the diversity of eastern and western cultures, in terms of thinking style, westerners focus on rational, analytical and empirical, analyzing the overall integration as a whole, however, Chinese focus on intuitive, holistic and experience. So the word “nature” in item #3 “I understand how the [intervention] affects the nature of my own work” was too abstract to understand for Chinese. There were positive relationships between the part C four constructs score and part B three general questions score, which was consistent with the other language version.

In the Chinese version of NoMAD, we integrated the four constructs as a whole and renumbered item #1–19, not separate the part C into four sections, then we add explanations to the survey instructions “item#1–3 measure Coherence; item #4–7 measure Cognitive Participation; item #8–14 measure Collective Action and item #15-#19 measure Reflexive Monitoring”.

The Chinese version of NoMAD has 22 items with four dimensions, which is consistent with the structure of the normalization process theory. The tool has moderate items, easy to understand, simple to use, and generally takes 2 ~ 5 min to complete the test, which has good operability. When using the C-NoMAD, there are some suggestions. Firstly, because the part B three general questions are similar, it is necessary to highlight the different words such as “familiar” “is currently” and “will become” to remind respondents to pay attention. Secondly, in part C, in order to easy to understand for respondents, some expressions should be adjusted tailored to the specific situation, for example, item #2 “staff in this organisation” in our study should be adjusted to “nurses in our hospital”; item #9 “working relationships” should be adjusted into “nurse-to-patient relationships”; item #16 “the staff” should be adjusted into “nurses”.

In addition, the open-ended questions’ results about the participants’ comments on PI management system suggestions also provided new insight. The NoMAD is a structured measurement tool, which can quantify implementation problems. However, quantitative data can help us investigate the current implementation process, while qualitative data can help us explore its deeper reason. The qualitative feedback would guide us to better improve our implementation interventions and strategies. When using the NoMAD tool in the future, we recommend adding several open-ended questions related to the topic, which will be beneficial.

### Limitations

Due to human, material, and time restrictions, this study evaluated an innovative intervention in only one hospital, involving only nursing staff, so the representativeness of the results is limited. And the findings suggested deleting item #3, requiring future research to justify it in different populations, interventions, and institutions. Furthermore, this study only investigated once and did not conduct a continuous follow-up of this PI management system in the implementation process to explore the dynamic implementation process, resulting in the results only reflecting the performance at that time, so future research about comparing the changes of implementation sustainability at various time nodes is necessary.

## Conclusions

The Chinese version of NoMAD is a reliable and valid tool to evaluate the implementation process from the perspective of Chinese health professionals directly involved in the work of implementing complex interventions in the clinic. Simple and clear quantitative assessment tools can help clinical researchers, care managers, and policy makers identify facilitators and barriers when a new technology, approach, or complex intervention becomes routine in clinical practice. This study provides a basis for developing interventions that facilitate the clinical implementation of new technologies or approaches, and is important for accelerating the pace of biomedical research findings for human health.

## Data Availability

The dataset generated and analyzed during the current study are available from the first author or corresponding author on reasonable request.

## Abbreviations

CFI: Comparative Fit Index
CVI: Content validity index
I-CVI: Item-level CVI
NoMAD: Normalization MeAsure Development
NPT: Normalization Process Theory
PI: Pressure injuries
RMSEA: Root mean square of approximation
S-CVI: Scale-level CVI
SRMR: Standardized Root Mean Square Residual
TLI: Tucker Lewis Index
WLSMV: Weighted Least Square Means and Variances
